# Differential View on the Bile Acid Stress Response of *Clostridioides difficile*

**DOI:** 10.3389/fmicb.2019.00258

**Published:** 2019-02-18

**Authors:** Susanne Sievers, Nicole G. Metzendorf, Silvia Dittmann, Daniel Troitzsch, Viola Gast, Sophie Marlen Tröger, Christian Wolff, Daniela Zühlke, Claudia Hirschfeld, Rabea Schlüter, Katharina Riedel

**Affiliations:** ^1^Institute of Microbiology, Center for Functional Genomics of Microbes, University of Greifswald, Greifswald, Germany; ^2^Department of Microbial Proteomics, Institute of Microbiology, Center for Functional Genomics of Microbes, University of Greifswald, Greifswald, Germany; ^3^Imaging Center of the Department of Biology, University of Greifswald, Greifswald, Germany

**Keywords:** *Clostridioides difficile*, proteomics, bile acids, motility, flagella, stress response, chaperones

## Abstract

*Clostridioides difficile* is an intestinal human pathogen that uses the opportunity of a depleted microbiota to cause an infection. It is known, that the composition of the intestinal bile acid cocktail has a great impact on the susceptibility toward a *C. difficile* infection. However, the specific response of growing *C. difficile* cells to diverse bile acids on the molecular level has not been described yet. In this study, we recorded proteome signatures of shock and long-term (LT) stress with the four main bile acids cholic acid (CA), chenodeoxycholic acid (CDCA), deoxycholic acid (DCA), and lithocholic acid (LCA). A general overlapping response to all tested bile acids could be determined particularly in shock experiments which appears plausible in the light of their common steroid structure. However, during LT stress several proteins showed an altered abundance in the presence of only a single or a few of the bile acids indicating the existence of specific adaptation mechanisms. Our results point at a differential induction of the groEL and dnaKJgrpE chaperone systems, both belonging to the class I heat shock genes. Additionally, central metabolic pathways involving butyrate fermentation and the reductive Stickland fermentation of leucine were effected, although CA caused a proteome signature different from the other three bile acids. Furthermore, quantitative proteomics revealed a loss of flagellar proteins in LT stress with LCA. The absence of flagella could be substantiated by electron microscopy which also indicated less flagellated cells in the presence of DCA and CDCA and no influence on flagella formation by CA. Our data break down the bile acid stress response of *C. difficile* into a general and a specific adaptation. The latter cannot simply be divided into a response to primary and secondary bile acids, but rather reflects a complex and variable adaptation process enabling *C. difficile* to survive and to cause an infection in the intestinal tract.

## Introduction

The anaerobic bacterium *Clostridioides difficile* represents one of the most serious nosocomial pathogens and is the main cause of antibiotics-associated diarrhea (Thomas et al., 2003). Two main toxins (Toxins A and B) provoke a disruption of the intestinal epithelium and a strong inflammatory host response leading to symptoms from mild diarrhea to more serious and often life-threatening conditions such as pseudomembranous colitis, toxic megacolon and eventually an intestinal perforation (Bartlett, 2006; Rupnik et al., 2009). The expression level of toxins in *C. difficile* was shown to be not only strain dependent, but also tightly connected to the growth state and basic physiology of the bacterium (Karlsson et al., 2008; Martin-Verstraete et al., 2016).

**FIGURE 1:**
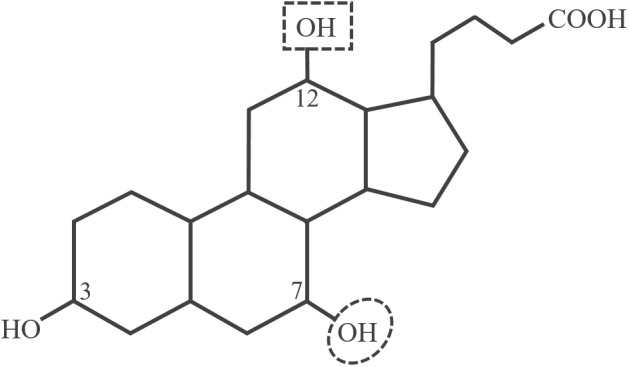
Structure of unconjugated cholic acid (CA). The encircled hydroxy group on C7 is missing in the secondary bile acids deoxycholic acid (DCA) and lithocholic acid (LCA). Chenodeoxycholic acid (CDCA) and LCA do not possess the C12 hydroxy group.

As an intestinal pathogen *C. difficile* has to deal with high concentrations of different bile acids, amphiphilic substances with a steroid nucleus (Figure 1). Bile acids are produced by the liver in order to facilitate absorption and digestion of dietary lipids. Due to their soap-like character, bile acids act as natural antimicrobials and only organisms adapted to the challenge will survive in the intestines (Begley et al., 2005). The two main bile acids produced in the human liver are cholic acid (CA) and chenodeoxycholic acid (CDCA) mostly conjugated to taurine or glycine. Species of the intestinal microbiota are capable of deconjugating the primary bile acids, and by dehydroxylation at C7 they can convert CA and CDCA to secondary bile acids resulting in deoxycholic acid (DCA) and lithocholic acid (LCA), respectively (Figure 1). Thus, the microbiota largely contributes to the shaping of the intestinal bile acid composition (Long et al., 2017).

Thirty-five years ago, it was described that bile acid preparations can stimulate the germination of *C. difficile* spores (Wilson, 1983). However, it took another 25 years until Sorg and Sonenshein (2008) elucidated CA as the active component of bile to instigate germination. Not much later they discovered an inhibitory effect of CDCA and analogs of it on *C. difficile* spore germination (Sorg and Sonenshein, 2009). In 2013, Francis et al. identified the receptor CspC on the *C. difficile* spore that directly interacts with CA to initiate germination (Francis et al., 2013). Hitherto, no further direct protein-bile acid interactions in *C. difficile* have been described. However, interesting findings on a negative effect of bile acids on the action of *C. difficile* toxins point at a possible direct interaction of the two (Brandes et al., 2012; Darkoh et al., 2013).

Besides the positive effect of CA on spore germination, an inhibitory effect of secondary bile acids not only on germination but also on growth and virulence of *C. difficile* has been frequently described (Lewis et al., 2016; Winston and Theriot, 2016; Thanissery et al., 2017). In light of this, the association of a depleted microbiota, which involves an increased ratio of primary to secondary bile acids, and the increased susceptibility to a *C. difficile* infection becomes evident. Very recently, Lewis et al. (2017) could even show that *C. difficile* strains with a higher tolerance for secondary bile acids exhibit a greater disease severity in mice and humans.

During the last years, research focused on unraveling the enzymatic and metabolic crosstalk between species of the microbiota and *C. difficile* which already led to the identification of some key players including their metabolic abilities in this complex network (Theriot et al., 2014; Buffie et al., 2015; Greathouse et al., 2015; Ridlon et al., 2016; Theriot et al., 2016; Yoon et al., 2017). The gained knowledge is a starting point for the development of personalized and disease-specific strategies to manipulate a patient’s intestinal microbiota in the most favorable way (Forster and Lawley, 2015) and to provide an alternative treatment option to the successful but still not fully understood fecal microbiota transplantation (Weingarden et al., 2014; Seekatz et al., 2018).

The microbiota must be studied as a complex system, and certainly the single bricks of this entity influence and depend on each other. Still, in order to fully understand the processes and dependencies within the highly complex intestinal microbial community, single species including their gene expression, physiological abilities and responses upon stimuli have to be investigated (Theriot, 2018).

In this study, we aimed at the characterization of *C. difficile*’s stress response to the four main human bile acids. Although, a phenotypic description of growth differences upon challenge with different bile acids has been published (Lewis et al., 2016; Thanissery et al., 2017), no information on the adaptation of gene expression is available until now. In this study, a comprehensive proteomics approach to record stress signatures of the unconjugated bile acids CA, CDCA, DCA and LCA in shock experiments as well as during LT stress conditions has been employed revealing a general stress response to all four bile acids, but also specific responses to only one or a few of the different bile acids.

The specific bile acid compositions of patients may affect germination and growth of *C. difficile* differently. Knowledge on the specific responses of *C. difficile* to different bile acids and on bile acid abundance in a patient could allow speculation on the susceptibility to *C. difficile* and the course of an infection.

## Materials and Methods

### Strains, Media and Growth Conditions

For bile acids shock experiments *C. difficile* 630Δerm (Hussain et al., 2005) was inoculated to an A_600_ of 0.05 and grown anaerobically at 37°C in brain heart infusion medium (BHI, Oxoid). At an A_600_ of 0.4 the cultures were shocked with sub-lethal concentrations of the sodium salts of cholic acid [6 mM CA (Sigma-Aldrich)], chenodeoxycholic acid [0.6 mM CDCA, (Sigma-Aldrich)], deoxycholic acid [0.6 mM DCA, (Sigma-Aldrich)], and lithocholic acid [0.08 mM LCA, (Steraloids Inc., RI, United States)] finally in solution, or left untreated. After 90 min, cells were harvested anaerobically on ice, pelleted by centrifugation and washed twice in ice-cold, oxygen-free PBS (pH 7.2) buffer. In long-term (LT)-stress experiments *C. difficile* 630Δerm was inoculated to an A_600_ of 0.01 in BHI and grown in the presence of varying concentrations of each of the four bile salts over a period of 18 h. Bile acid stressed *C. difficile* cells and unstressed cells were sampled in exponential growth phase (15 mM CA at *A*_600_ = 0.3, 0.8 mM CDCA at *A*_600_ = 0.3, 0.8 mM DCA at *A*_600_ = 0.3, 0.08 mM LCA at *A*_600_ = 0.8 and untreated at *A*_600_ = 0.8), pelleted and washed as previously described (Sievers et al., 2018). Although sodium salts of the bile acids were used in this study, they are referred to as bile acids throughout the manuscript.

### Protein Extraction and MS Sample Preparation

Cell lysis and acetone precipitation of proteins was carried out as previously described (Otto et al., 2016). In brief, cell pellets were resuspended in 50 mM TrisHCl, pH 8 containing 8 M urea, 3 M thiourea, 10 mM EDTA, 4% CHAPS and 20 mM Tris (2-carboxyethyl)phosphine (TCEP). Cells were lysed by 6 cycles of 1 min intervals of ultrasonication (Sonotrode MS73, Bandelin, Germany) with intermittent cooling. Cell debris was removed by centrifugation, supernatants mixed with water 1:2 and proteins precipitated overnight at −20°C by adding 4 volumes of acetone. Protein pellets were resuspended in 50 mM TEAB (Triethylammonium bicarbonate) containing 0.1% (w/v) RapiGest SF (Waters, United States) and the protein concentration determined using Roti Nanoquant (Carl Roth, Germany). 500 μg of protein were reduced in 100 μl 50 mM TEAB, 0.1% RapiGest, 5 mM TCEP for 45 min at 60°C. Afterward free thiols were alkylated for 15 min in the dark with 10 mM iodoacetamide. For an in-solution digestion of proteins, trypsin was added in a ratio of 1:100 and digests incubated in a thermo mixer for 5 h at 37°C and 900 rpm. Digestion was stopped and RapiGest removed as recommended by the manufacturer. Sample Peptides were cleaned via StageTipping as described elsewhere (Rappsilber et al., 2007). Purified peptides were dissolved in 100 μl of 2% (v/v) acetonitrile, 0.1% (v/v) acetic acid containing 50 fmol/μl spiked-in yeast alcohol dehydrogenase (Waters).

### LC-MS^E^ Analysis

Peptides were analyzed using a nanoACQUITY^TM^ UPLC^TM^ system (Waters) coupled to a Synapt G2 mass spectrometer (Waters). Details on liquid chromatography and IMS^E^ (MS^E^ with ion mobility separation) methods are described elsewhere (Muntel et al., 2014; Zuhlke et al., 2016). LC-IMS^E^ data were processed using PLGS v3.0.1. Processing parameters were applied according to Schmidt et al. (2017).

MS^E^ raw data were searched against the randomized *C. difficile* 630 Δerm database published by Dannheim et al. (2017) containing 3781 protein entries of *C. difficile* plus laboratory contaminants and the yeast ADH1 sequence. For a positive protein identification the following criteria had to be met: 1 fragment ion matched per peptide, 5 fragment ions matched per protein, 1 peptide matched per protein; 2 missed cleavages allowed, primary digest reagent: trypsin, fixed modification: carbamidomethylation C (+57.0215), variable modifications: deamidation N and Q, oxidation M, pyrrolidonecarboxylacid N-TERM. The protein false discovery rate (FDR) was set to 5% and only identifications based on at least two peptides were considered in the final analysis. Three biological replicates, each run in three technical replicates were conducted for shock experiments. LT stress experiments were performed in 4 biological replicates with three technical replicates of each.

### Protein Quantification, Data Evaluation and Visualization

Data on protein quantities were corrected for detector saturation effects by implementing a correction factor as recently described (Zuhlke et al., 2016). Datasets were normalized on the basis of the spiked-in yeast alcohol dehydrogenase. The mass spectrometry proteomic data have been deposited in the ProteomeXchange Consortium via the PRIDE partner repository (Vizcaino et al., 2016). Results of the shock experiments can be found with dataset identifier PXD010514. LT stress data are linked with identifier PXD010525. The Perseus software platform (Tyanova et al., 2016) was used for the further analysis of proteomic datasets as recently described (Neumann-Schaal et al., 2018). Data were filtered (shock experiments: 3 out of 3 replicates and LT stress experiments: 4 out of 4 replicates within one group of valid values), log2 transformed and normalized by division by the means to generate volcano plots as well as to carry out ANOVA testing (permutation-based, FDR 0.01 for shock and 0.001 for LT stress experiments) to create heatmaps (*z*-score normalization, distance euclidean). For principal component analysis (PCA) missing data was imputed (settings: width 0.3, down shift 1.8, for PCA: cut off Benjamini Hochberg FDR 0.05). Interactive 3D plots were generated using the Plotly package in R v. 3.4.3 (30 November, 2017), (Sievert et al., 2016). Voronoi treemaps were built using the Paver software (DECODON GmbH) as previously described (Otto et al., 2016).

### Motility Assay

The influence of bile acids on the swimming motility of *C. difficile* was tested on 10 cm diameter soft agar plates (BHI with 0.175% [w/vol] agar). A sigH mutant was used as a non-motile control (Saujet et al., 2011). Plates were inoculated with single colonies that were pierced onto the centers of the plates with increasing concentrations of bile acids. Plates were incubated anaerobically at 37°C. Diameters of halos due to bacterial migration were measured 48 h after inoculation.

### Electron Microscopy and Flagella Quantification

Bile acid conditions and time points of harvest for negative staining were chosen as in LT stress experiments. *C. difficile* was pelleted by centrifugation (4,000 × g, 4°C, 10 min). Cells were washed once with sterile-filtered (0.2 μm) 1 × PBS and fixed (2.5% glutaraldehyde, 2% paraformaldehyde in PBS) for 1 h at RT followed by at least 12 h at 4°C. Samples were centrifuged and washed three times with sterile-filtered 1 × PBS. Pellets were resuspended in sterile-filtered 1 × PBS. The flotation method was used for the negative staining procedure. Fixed cells were allowed to adsorb onto a glow-discharged Pioloform carbon-coated 400-mesh grid for 10 min. The grid was then transferred onto two droplets of deionized water, and finally onto a drop of 0.5% aqueous uranyl acetate for 10 s. After blotting with filter paper and air-drying, the samples were examined with a transmission electron microscope LEO 906 (Carl Zeiss Microscopy GmbH) at an acceleration voltage of 80 kV. Sharpness and contrast of micrographs were adjusted by using Adobe Photoshop CS6. Transmission electron micrographs at a 1293-fold magnification were analyzed with Fiji (Schindelin et al., 2012) to determine the length and width of the cells as well as the total length of all visible flagella. For the investigation of the area covered with flagella, a grate with squares (1.6 μm^2^) was applied to all images. Squares with flagella were counted as positive. All data were further analyzed using Microsoft Excel. Statistical significance was assessed by an unpaired Student’s *t*-Test.

## Results

### Growth-Inhibiting Effect of Bile Acids

To determine inhibitory concentrations of bile acids in *C. difficile* two different types of stress experiments were conducted. First, sub-lethal concentrations of the four different bile acids were determined in shock experiments, i e., exponentially growing cells were shocked with concentrations that prevented further growth of *C. difficile*, but did not cause lysis. Sub-lethal concentrations of CA, DCA and CDCA were defined as 6, 0.6 and 0.6 mM, respectively. Thus, CA could be added in an amount 10 times higher compared to DCA and CDCA until causing the same effect on growth. LCA was used at a maximum concentration of 0.08 mM. This caused only a slight decrement of growth, but higher concentrations of LCA resulted in formation of micelles and thereby impaired measurements of the optical density.

In a second experimental setup *C. difficile* was subjected to long-term bile acid stress. To this end, the bacterium was inoculated with a low starting A_600_ of 0.01 into medium already containing the bile acids. Different concentrations of the single bile acids were tested and growth was measured over a period of 20 h until cultures reached stationary phase to identify concentrations of each bile acid that cause a strong and comparably inhibitory effect allowing the cultures to grow up to an A_600_ of about 1 (Supplementary Figure S1). LCA was again used at the maximum possible concentration of 0.08 mM which had only a minor effect on the growth of *C. difficile* compared to control conditions. The other three bile acids were applied in concentrations considerably higher than the sub-lethal ones used in shock experiments pointing at an ability of *C. difficile* to adapt to the presence of bile acids. Also in LT stress experiments, much higher concentrations of CA were tolerated compared to DCA and CDCA. However, *C. difficile* cells grown in presence of CA showed a strikingly longer lag-phase compared to the DCA and CDCA-treated cultures (Supplementary Figure S1).

### Differential Response to Bile Acids

#### Shock Response

**FIGURE 2:**
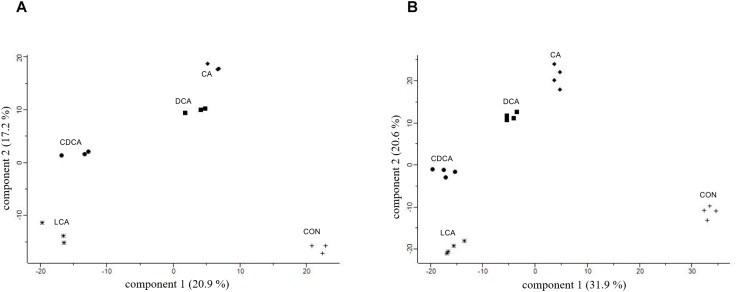
Global proteomic datasets of shock experiments **(A)** and LT stress **(B)** depicted in PCAs. Bile acids are abbreviated as follows: CA, cholic acid; DCA, deoxycholic acid; CDCA, chenodeoxycholic acid; LCA, lithocholic acid; CON, unstressed culture.

For comparative proteome analyses, *C. difficile* cultures shocked with sub-lethal concentrations of bile acids determined above were harvested together with an untreated control culture after 90 min of shock onset. The *C. difficile* culture shocked with 0.08 mM LCA kept growing almost to the same extent as the control. Still, in a principal component analysis (PCA) comprising all three replicates of all five conditions, the control samples clearly separate from LCA stress and all other stresses and CA and DCA cluster together apart from CDCA and LCA (Figure 2A). Hence, a primary bile acid clusters closer with its corresponding secondary bile acid than with the other primary bile acid.

#### LT Stress Response

For comparative proteome analyses, *C. difficile* cells were constantly grown in absence (controls) and presence of bile acids, and samples for comprehensive proteome analyses were taken in the late-exponential growth phase in four replicates for each condition (15 mM CA, 0.8 mM CDCA, 0.8 mM DCA, 0.08 mM LCA). As observed in shock experiments, the expression profiles of the control cultures clearly differ from those of bile acid-stressed cultures. However, these LT stress experiments also clearly delineate the changes in the expression profiles of the different bile acids, whereas biological replicates correspond very well (Figure 2B). Since all cells were harvested during late-exponential growth, the observed differences in the protein profiles correspond to specific stress responses caused by each of the tested bile acids.

### Bile Acids Induce a General and Specific Response

#### Proteome Shock Signatures

**FIGURE 3:**
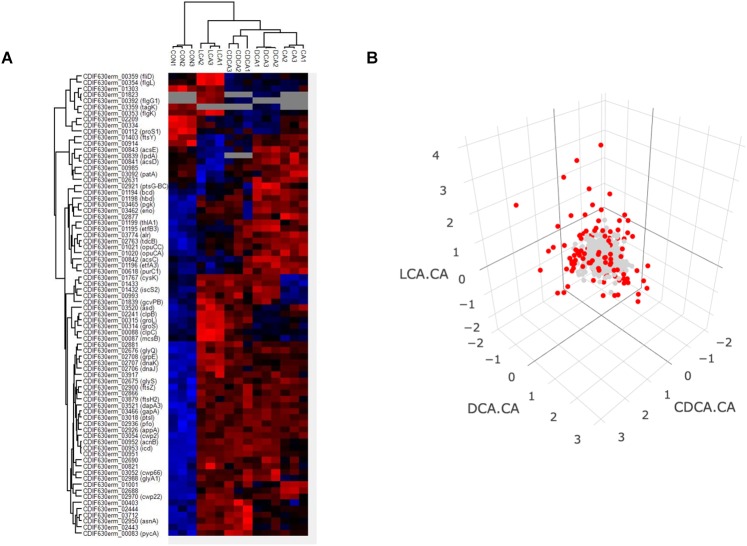
Heatmap comprising all three replicates of control and the four bile acid samples **(A)**. Proteins which were not quantified in all three replicates of a specific condition were not considered indicated by gray coloring. Red and blue colors indicate high and low protein abundance, respectively. A 3-D plot of ratios of protein abundance [Δlog2(LCA-CA), Δlog2(CDCA-CA), Δlog2(DCA-CA)] for proteins which could be quantified in all four different bile shock conditions is shown **(B)**. Protein dots for which the log2 ratio is higher than 1 or below –1 are shown in red (FC ≥ 2). A rotatable 3D plot is provided supplementary (Supplementary Figure S3).

Proteins that were quantified in 3 out of 3 replicates were subjected to an ANOVA analysis to identify differentially expressed proteins comparing bile acid conditions with control but also bile acid conditions among each other (Supplementary Tables S1, S2). 76 proteins met the criterion of a permutation based FDR of 0.01 and thus appeared to be differentially abundant between the tested conditions (Figure 3A). Approximately one third of these proteins represent a general bile acid stress response, i.e., their abundance proved to be higher in presence of each of the tested bile acids compared to control conditions. Amongst these are chaperones DnaK/DnaJ/GrpE, cell wall binding proteins (Cwp2, Cwp66, Cwp22) and proteins involved in cell division (FtsZ, FtsH2). Notably, the abundance of several proteins differed significantly depending on the kind of bile acid used in the shock experiment indicating bile acid-specific responses. To better identify these differentially expressed proteins, protein amounts of each condition were compared to every other condition and visualized in volcano plots and Voronoi treemaps (Supplementary Figure S2). Furthermore, ratios of LCA vs. CA, DCA vs. CA and CDCA vs. CA were calculated and visualized in a 3D-plot (Figure 3B). Data points close to the origin represent proteins similarly expressed amongst different bile acids, but the longer the distance between data point and origin, the more distinctive is the response to a specific bile acid. An animated and rotatable version of Figure 3B is provided supplementary (Supplementary Figure S3). Surprisingly, and in contrast to the DnaK/DnaJ/GrpE system, the chaperonins GroL and GroS as well as chaperone ClpB, the ATPase ClpC and the associated arginine kinase McsB are not generally induced by bile acids. These proteins highly increase in amount after shock with LCA and extenuated after CDCA shock, but were not affected by DCA or CA. Moreover, LCA, DCA and CDCA, but not CA seem to interfere with the synthesis of iron sulfur clusters, since protein CysK, which catalyzes the formation of cysteine from O-acetyl-serine and H_2_S, and proteins IscS2, a cysteine desulfurase, and Fe-S cluster assembly protein CDIF630erm_01433 are all higher abundant compared to control conditions. In summary, we identified numerous proteins, expression of which is similarly altered in response to all tested bile acids, but also a significant number of proteins, whose expression seems to be specifically affected by individual bile acids.

#### Proteome LT Stress Signatures

**FIGURE 4:**
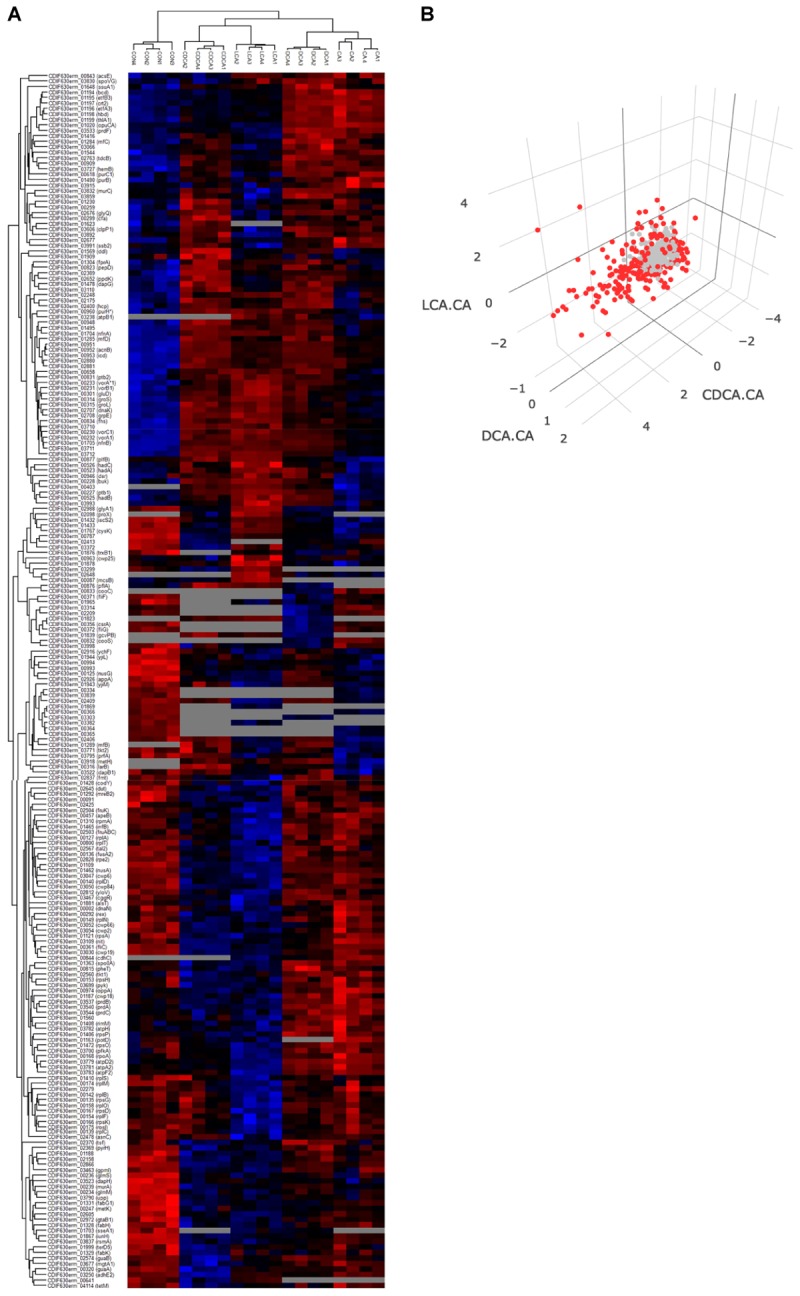
Four replicates of control and the four bile acid samples are visualized in a heatmap **(A)**. Only proteins quantified in all replicates of a specific condition were considered, otherwise they were grayed out. Higher and lower protein abundance is indicated in red and blue colors, respectively. For proteins that could be quantitated in all four bile acid stress conditions, ratios of protein abundance [Δlog2(LCA-CA), Δlog2(CDCA-CA), Δlog2(DCA-CA)] were calculated and visualized in a 3D-plot **(B)**. Red data points symbolize proteins of a log2 ratio higher than 1 or below –1. In a supplementary version of Figure 4B the perspective can be rotated (Supplementary Figure S3).

For the evaluation of the LT bile acid stress experiment, proteins quantified in 4 out of 4 replicates were chosen for an ANOVA analysis (Supplementary Tables S3, S4). Applying a very stringent and permutation-based FDR of 0.001 still yielded 222 proteins differing in abundance when cells were grown in the absence and presence of different bile acids (Figure 4A).

**FIGURE 5:**
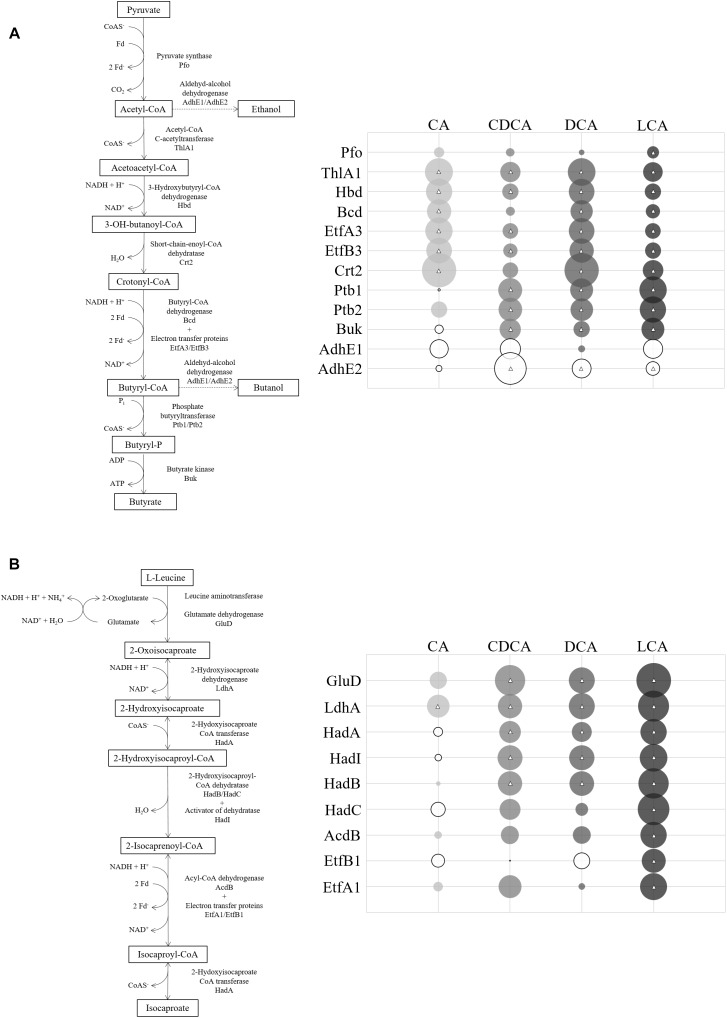
**(A)** Fermentation pathway of pyruvate down to butyrate with branches to ethanol and butanol. The abundance of corresponding enzymes in relation to control conditions is symbolized in circles. Size of circles corresponds to extent of fold change (FC). Solid circles represent FCs bigger than one (bile acid/control) and open circles represent FCs less than one. Circles centered with a triangle are based on a quantitation with *p*-values < 0.05. **(B)** Reductive Stickland reaction of leucine to isocaproate (adapted from Kim et al., 2005) with quantitative proteomic data of enzymes depicted in circles. Exact values of protein quantification and *p*-values are provided in Supplementary Table S4.

In contrast to the shock experiments, there was no extensive general bile acids stress response with similar changes in protein abundance across all four LT stress conditions. A central metabolic pathway in *C. difficile* that was affected during LT challenge is the conversion of pyruvate to fermentation products as ethanol, butanol or butanoate (Hofmann et al., 2018). Enzymes of this pathway were quantitatively affected by all tested bile acids, however, not always uniformly or to the same extent (Figure 5A). Apparently, all bile acids provoke a decrease of alcohol dehydrogenases AdhE1 and AdhE2 and thereby hamper the conversion of acetyl-CoA to ethanol or butanol. Enzymes of upstream reactions catalyzing the conversion of acetyl-CoA to butanoyl-CoA are mostly up-regulated by CA and DCA, but enzymes involved in the processing of butanoyl-CoA to butanoate are up-regulated only in the presence of CDCA, DCA and LCA, but not CA compared to control conditions. Numerous other proteins are more abundant when cells were subjected to continuous stress of the different bile acids with exception of CA (Figure 4A), e.g., VorAA^∗^BC1 involved in the oxidative Stickland reaction of branched chain amino acids as well as proteins belonging to the Gro and Dna chaperone machineries, which contrasts with the results of the shock experiment. Moreover, almost all enzymes of the reductive Stickland fermentation of leucine to isocaproate, representing another key metabolic pathway in *C. difficile* (Hofmann et al., 2018), were found to be induced by all bile acids except CA (Figure 5B).

Almost one third of the 222 differentially expressed proteins follow the pattern of a significant downregulation in LCA and CDCA stressed cells vs. DCA, CA and control conditions. Representative proteins of this cluster are the subunits A2, D2, H, and F2 of the ATP-Synthase complex. Also, several sub-units building the proline reductase complex (PrdBAC) are more abundant at CA and DCA compared to LCA and CDCA. However, the major fraction of gene products with such specific regulation pattern is represented by cell wall-bound proteins (Cwp2/6/18/19/66/84) and proteins known to be involved in the formation of the cell wall (MurA, MreB1, and B2).

As for shock datasets, data of the long-term stress experiments were also compared pairwise in volcano plots and Voronoi treemaps (Supplementary Figure S4) and the ratios of LCA vs. CA, DCA vs. CA and CDCA vs. CA visualized (Figure 4B). This 3D-plot, whose perspective can be conveniently rotated (Supplementary Figure S3), emphasizes the high number of proteins that are subject to a specific regulation during LT bile acid challenge.

### Changes in Morphology

**FIGURE 6:**
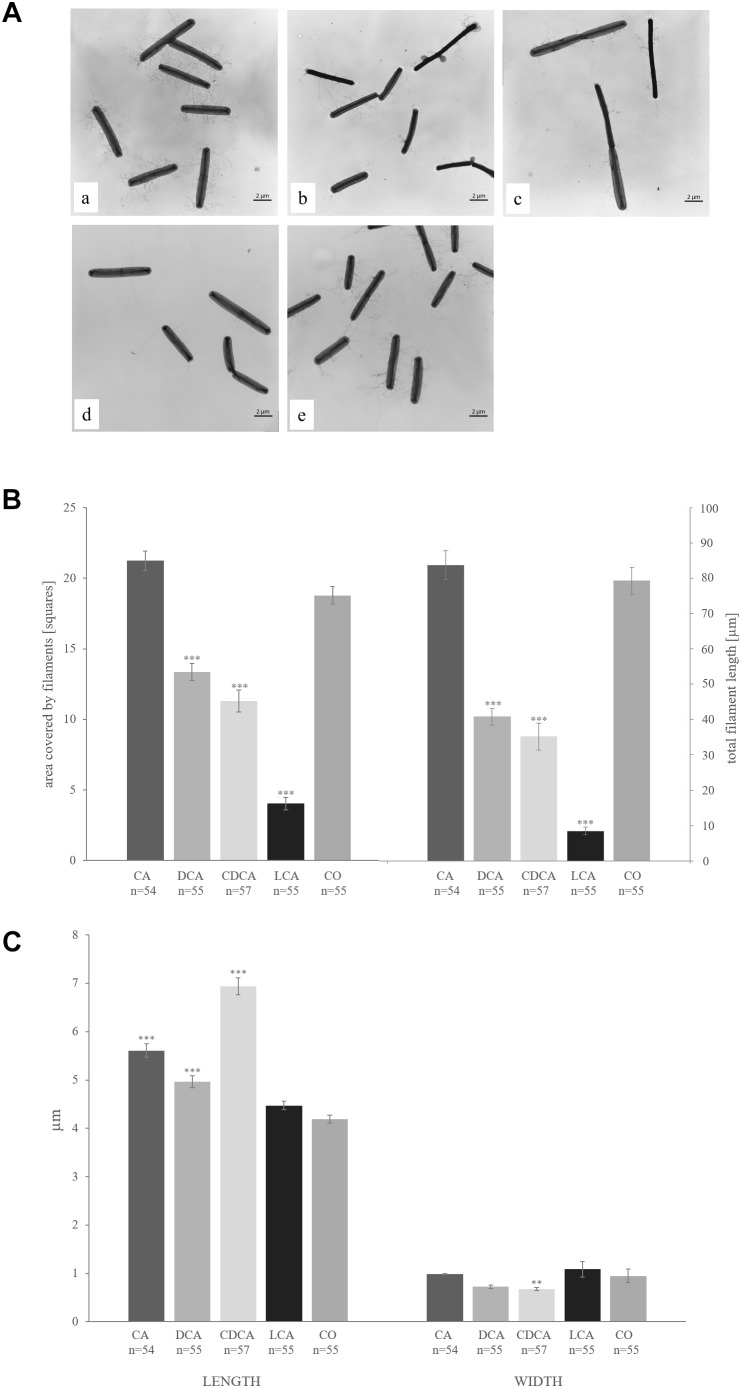
**(A)** Transmission electron micrographs of *C. difficile* challenged with the four different bile acids CA **(a)**, DCA **(b)**, CDCA **(c)**, LCA **(d)** and unstressed cells **(e)**. **(B)** The covered area (left columns) and the total length (right columns) of flagella per cell were measured and are given in squares und μm, respectively. **(C)** Average length (left columns) and width (right columns) of bacterial cells are given in μm. Values significantly different in the presence of bile acid compared to non-treated cells (CO) are marked (^∗∗∗^*p*-value < 0.001; ^∗∗^*p*-value < 0.01).

The proteome analysis of LT-stressed cells revealed a dramatic decrease of the major structural flagellum protein FliC in the presence of CDCA and LCA compared to control conditions. Also DCA stress resulted in down-regulation of FliC, but not as dramatically. Interestingly, CA did not provoke any altered fliC expression. Another 22 proteins of the flagellar filament have been identified in this proteome analysis (from gene loci CDIF630erm_00349 down to CDIF630erm_00395), but none of them could be identified in all bile acids stress conditions. Strikingly, 15 of these proteins were identified in all four replicates of control and CA conditions, but not in a single replicate of the other three bile acid stress conditions (Supplementary Table S3). Thus, the expression of these proteins appears to be entirely switched off in the presence of selected bile acids. This observation prompted us to test for the presence of flagella in cells grown in presence of the different bile acids by electron microscopy (Figure 6A). *C. difficile* is extensively flagellated under control conditions. Notably, the electron micrographs of bile acid-stressed cells correlate very well with protein quantification results. Cells challenged with CA are just as flagellated as unstressed cells, whereas fewer filaments have been observed on DCA- and CDCA- stressed *C. difficile*, and LCA basically provoked a complete loss of flagellar filaments. To quantify the presence of flagella, the length and the area covered by them were measured in more than 50 cells / condition in electron micrographs (Figure 6B). Besides the change in the number of flagella, electron micrographs also revealed an alteration in cell morphology. Whereas cells at control conditions are 4 μm long on average, DCA-, CA- and especially CDCA-stressed cells were found to be significantly longer with up to 7 μm (Figure 6C).

### Decrement of Motility

**FIGURE 7:**
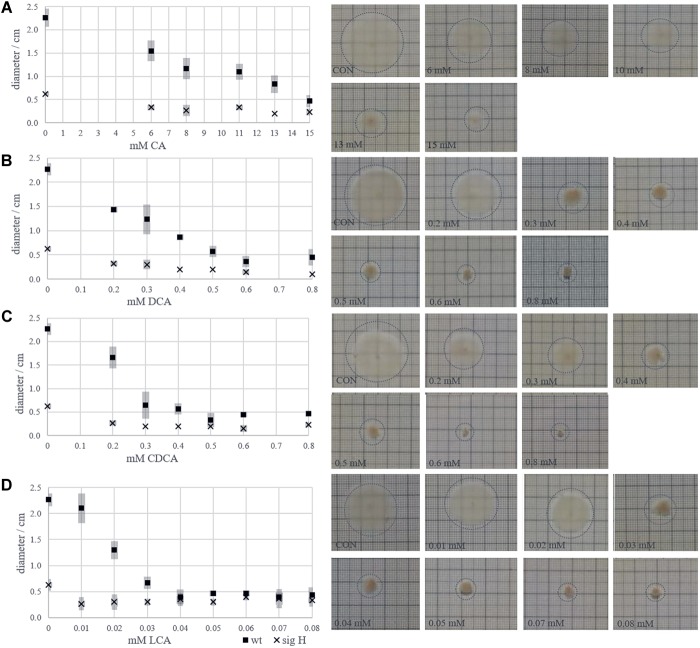
Zones of growth of *C. difficile* inoculated in the center of soft agar plates provided with increasing concentrations of bile acids. Diameters of growth halos in dependence of bile acid concentration are given in diagrams (left) accompanied by images of exemplary agar plates (right) for CA **(A)**, DCA **(B)**, CDCA **(C)**, and LCA **(D)**.

The bile acid-specific loss of flagella should lead to a reduced motility of *C. difficile*. Therefore, the motility of the bacterium with increasing concentrations of each bile acid was tested on soft agar plates. An immotile sigH mutant of *C. difficile* was used as a negative control. The maximum concentration of bile acids in the motility assays was chosen up to the growth inhibiting concentrations used in LT-stress experiments (15 mM CA, 0.8 mM CDCA, 0.8 mM DCA, 0.08 mM LCA). Figure 7A–D depict the decreasing motility of *C. difficile* 630Δerm, which is *per se* not very motile, with increasing concentrations of CA, DCA, CDCA, and LCA, respectively. However, a decreasing growth zone in response to the presence of bile acids might be the result of two superimposed effects. Increasing bile acid concentrations hamper growth in general, but could also specifically interfere with flagella-driven motility. The diameter of the growth zones of *C. difficile* challenged with CA and DCA decreased linearly with increasing concentrations. Presumably, this decrease is predominantly caused by the general inhibition of growth. However, the growth zone diameter seems to drop rapidly in presence of 0.3 mM CDCA and 0.03 mM LCA. These concentrations have only a minor (CDCA) or no effect (LCA) on growth in liquid cultures of *C. difficile* and might therefore rather impair motility than growth of *C. difficile*.

## Discussion

The amphiphilic nature of bile acids is challenging to bacterial cell integrity. At higher concentrations bile acids cause disruption of cellular membranes and subsequently cell lysis accounting for their antimicrobial activity. However, at sub-lethal concentrations bile acids might induce more specific effects on cells, e. g., a modulation of cellular signaling processes (Zhou et al., 2013). Notably, a ligand-receptor-like interaction has been described in *C. difficile* for taurocholic acid and the spore protein CspC, which initiates germination of the spore (Francis et al., 2013). The study presented herein aimed at the elucidation of so far unknown specific effects of selected bile acids going beyond a general detergent-like action.

As a starting point, different sub-lethal and inhibitory concentrations of the bile acids CA, DC, and CDCA were determined. While *C. difficile* tolerates high amounts of CA (6 mM in shock experiment), it appeared to be tenfold more sensitive to DC and CDCA. This might be due to the stronger hydrophobicity of the steroid scaffold of DCA and CDCA compared to CA featuring one additional hydroxy group which could decrease its cell-lysing capacity. It is also possible that fewer hydroxy groups facilitate the diffusion and uptake of bile acids into the cytoplasm where they can interfere with cellular processes. In this case, the tri-hydroxylated CA would mostly remain in the extracellular medium. In a follow-up study the extra- and intracellular bile acid concentrations after cell exposure will be determined by metabolic analyses of bile acids and derivatives of them. This will provide answers on how fast different bile acids enter *C. difficile* cells and if the bacterium is able to modify or even degrade them. Furthermore, an immunostaining of bile acids coupled to fluorescence- or electron microscopy will elucidate the location on the cellular surface or within the *C. difficile* cell and might disclose differences between the tested bile acids. Notably, when *C. difficile* was challenged with concentrations of bile acids higher than used in the shock experiments, cell lysis occurred. However, such concentrations could be employed for LT-stress experiments. This indicates that *C. difficile* is capable of adapting to bile acids by rearranging cellular structures to prevent cell lysis and even to grow in the presence of these high concentrations. In contrast to the other three bile acids, LCA could not be added to the medium in higher concentrations than 0.08 mM due to the formation of micelles. A conjugation of LCA with a small hydrophilic compound at the carboxy-group would have allowed a testing of higher concentrations, since the CMC (critical cellular concentration) would have been raised by the conjugation. However, in this study we decided to focus on the steroid scaffold as it occurs after deconjugation of the bile acids by microbes in the intestines and therefore used the sodium salts of unconjugated bile acids. Even though the LCA-concentration used in this study only had a small growth-inhibiting effect, quantitative proteomic data clearly indicated a stress response of *C. difficile* to this bile acid.

Our data indicate that a 90 min bile acid shock leads to a general cellular response independent from the chemical structure of the bile acids and likely due to their overall soap-like character. However, when *C. difficile* is continuously grown in presence of bile acids, thereby mimicking the *in vivo* conditions in the host intestines, numerous specific cellular adaptation processes but almost no general stress response could be observed.

The amphiphilic nature of bile acids also provokes an increasing denaturation of proteins leading to an up-regulation of chaperones in the cell. One interesting finding is the differential regulation of chaperones after bile acid shock. Whilst the expression of DnaK, DnaJ, and GrpE (encoded in one operon) increases in response to any of the tested bile acids, only LCA and less pronounced CDCA result in the induction of the chaperonins GroL and GroS, ClpB, the arginine kinase McsB and the ATPase ClpC, the latter two encoded in the operon also encoding the repressor protein CtsR. Although heat shock in *C. difficile* has been investigated yet (Ternan et al., 2012), the exact regulation of the different chaperones has not been described for *C. difficile* up to now. Notably, the respective coding operons in *C. difficile* are closely related to those of other well-studied bacteria, i.e., *Bacillus subtilis* (Schumann, 2016), and one would therefore predict a comparable regulation of gene expression. In *B. subtilis*, the *dna* and *gro* operons belong to the class I heat shock genes and are controlled by the transcriptional repressor HrcA. In *C. botulinum*, a derepression of the *dna* and *gro* operons in a *hrcA* mutant is reported, but the *gro* locus in that mutant is further inducible by heat, pointing at another factor of regulation (Selby et al., 2011). Possibly CtsR, the transcriptional repressor controlling the class III heat shock genes, could adopt this role, since Emerson et al. (2008) observed a similar regulation of typically CtsR regulated genes and *groLS* in microarray analyses, and our results of bile acid shock response also support this hypothesis. The here postulated double regulation of the *gro* locus by the repressors HrcA and CtsR has to be proven experimentally, but implies that all tested bile acids stimulate a HrcA response, whilst only LCA and CDCA induce a CtsR response.

Not only typical stress response proteins were up-regulated in the presence of bile acids, but also the abundance of enzymes involved in central metabolic pathways was altered. In the presence of bile acids, e. g., pyruvate was rather converted to butyric acid than to alcohols such as ethanol and butanol compared to non-stress conditions. Also, a strong induction of the reductive Stickland pathway converting L-leucine to isocaproate could be observed during LT-stress with DCA, CDCA, and LCA but remarkably not with CA. Several subunits of the ATP synthase dramatically decreased in amount in the LT-presence of CDCA and LCA, but not with CA and DCA. Likely, CDCA and LCA have a higher impact on membrane function and maintenance of the proton motive force (PMF) than DCA and CA resulting in dysregulation of enzymes that are involved in the generation of a proton gradient. The impact of bile acids on the PMF, and a possible difference depending on the sort of bile acid, will be tested by incubation of the cells with a carbocyanine-based dye whose fluorescence depends on the membrane potential. *C. difficile* possibly counteracts a decreased proton motive force and diminished ATP synthesis by an increased substrate level phosphorylation via the oxidation of pyruvate to acetyl-CoA for ADP phosphorylation. In this oxidation reaction ferredoxin is reduced and could be re-oxidized by the increase in leucine reduction. The higher demand of pyruvate is provided by pyruvate synthase. This enzyme contains iron sulfur clusters just as the HadBC proteins in the reductive Stickland reaction of leucine, which could be the reason for the enhanced synthesis of iron sulfur clusters during bile acids stress. This hypothesis needs to be verified in future experiments, in which the effect of surplus amounts of pyruvate and leucine on bile acids tolerance will be tested. Interestingly, also proteins of the D-proline reductase complex (PrdBAC) were determined with a strong decrease in abundance in the presence of CDCA and LCA. Kabisch et al. (1999) described the D-proline reductase in *Clostridium sticklandii* as a cytosolic selenoenzyme composed of three protein subunits. Future studies need to clarify if specific bile acids hamper formation or activity of D-proline reductase in *C. difficile*.

An important finding of this study is the differential effect bile acids have on the formation of flagella in *C. difficile*. Cells grown in the presence of CA synthesize flagella comparable to untreated cells. *C. difficile* stressed with the corresponding secondary bile acid DCA and the primary bile acid CDCA are characterized by fewer flagella, while cells challenged with LCA were basically completely impaired in flagella formation. A simple explanation of this observation would be that *C. difficile* is unable to build flagella because of a disturbed proton motif force or for the benefit of ATP saving. The impact of flagella on the motility, adherence and virulence for pathogenic bacteria, specifically for *C. difficile*, has been extensively discussed (Haiko and Westerlund-Wikstrom, 2013; Stevenson et al., 2015). It was shown that missing flagella, more specifically missing of the major structural component FliC, cause non-motility, but increase the adherence capability of *C. difficile* (Dingle et al., 2011) leading to an increased virulence in hamsters (Dingle et al., 2011) and mice (Barketi-Klai et al., 2014). The higher virulence could also be the consequence of a reported up-regulation of toxin synthesis in *fliC* mutated *C. difficile* cells (Aubry et al., 2012). Thus, the presence or absence of the major filament protein FliC does not only influence virulence directly by an altered motility and adhesiveness, but also has an impact on gene expression, e. g., the transcription of the pathogenicity locus encoding toxins A and B (Aubry et al., 2012; Barketi-Klai et al., 2014). Furthermore, due to its immunogenicity (Bruxelle et al., 2017), an absence of FliC could be advantageous during infection and help to evade the immune system. Our observation that the tested bile acids with exception of CA decrease flagella formation of *C. difficile* is thus highly relevant for the infection course. Bacteria in the upper intestinal tract face high CA concentrations, which facilitate spore germination and allow for flagellated and mobile vegetative cells. Along the intestines, the concentrations of secondary bile acids such as LCA increase leading to less flagellated bacteria that are still capable to adhere to tissue and might produce more toxins than in the small intestine or upper colon sections. Still the question remains, which regulatory mechanism leads to the absence of flagella during LCA stress but persistence of flagella even with very high levels of CA. It might simply be the stronger hydrophobicity of the steroid scaffold of LCA compared to CA that interferes with a proper assembly of flagella. However, it could also be speculated that LCA, as the most lipophilic of the tested bile acids, easily crosses the membrane and interferes intracellularly with regulatory processes controlling flagella formation, e. g., with a recently described flagellar switch (Anjuwon-Foster and Tamayo, 2017). Previously, Jain et al. investigated *C. difficile* cells mutated in the *dnaK* gene and found them to lack flagella (Jain et al., 2017), to be non-motile and longer than wild type cells. This phenotype very much resembles the one of some of the LT bile acid stressed cells of this study. Even though *dnaK* expression increased in bile acid stressed cells, the higher demand of DnaK possibly results in a lack of the chaperone for its regular functions as a putative role in flagella formation (Jain et al., 2017) and cell division. Another indicator of a disturbed cell division and cell wall formation in stressed cells is the observed dysregulation of several proteins involved in peptidoglycan synthesis (MurA, MreB1 and B2) and of several cell wall-bound proteins of mostly unknown function (Cwp2/6/18/19/66/84).

The bile acids tested in this study all carry their hydroxy groups in alpha conformation as they naturally occur in the human intestines. Thus, the hydroxy groups are located on one side of the steroid skeleton resulting in a hydrophobic plane on the opposite side. Modifications of the 7-OH group of bile acids as epimerization to beta conformation (Setoguchi et al., 1984; Lepercq et al., 2004), oxidation to a keto group (Macdonald and Hutchison, 1982) or dehydroxylation (Buffie et al., 2015) have been numerously reported. Whereas an epimerization of bile acids would bring hydroxy groups onto the opposite side of the steroid scaffold and thus decrease hydrophobicity, the dehydroxylation would increase hydrophobicity and thereby the toxic character of the bile acid (Hino et al., 2001). The formation of epimers and keto groups can compete with dehydroxylation (Macdonald and Hutchison, 1982) and could prevent formation of the secondary bile acids DCA and LCA from CA and CDCA, respectively, which could have a major impact on the outcome of a *C. difficile* infection. With respect to the protective nature of the microbiota during *C. difficile* infections, it would therefore not only be beneficial to feature bacteria as *C. scindens* that can convert primary to secondary bile acids (Buffie et al., 2015), but also to avoid alternative modifications of the hydroxy group on C7 competing with the dehydroxylation reaction. *C. difficile* itself is not capable of a dehydroxylation on C7, but it is not clear if it could alternatively modify the C7 hydroxy group to protect from the harmful action of secondary bile acids. Altogether, multiple modifications are possible and reported on C7 of the steroid scaffold, whereas the hydroxy group on C12 seems to be more static and difficult to modify. Indeed, a large cluster of proteins was similar regulated in CA and DCA opposed to CDCA and LCA LT-stress, respectively. The first two bile acids feature a C12 hydroxy group, which is missing in the latter two. The presence of the C12 hydroxy group on a specific bile acid could therefore be an important determinant for the action of this bile acid.

Previous studies on the effect of bile acids on *C. difficile* mostly concentrate on the benefit of taurocholic acid for the germination of spores or generally describe a growth inhibiting effect of secondary bile acids on vegetative cells. Proteomics data obtained in this study clearly showed that there is not only a general stress response of *C. difficile* to any of the tested bile acids, but there are also specific responses depending on the kind of bile acid. Our observations do not support the idea of a simple grouping into “adverse” primary and “beneficial” secondary bile acids concerning a *C. difficile* infection, but rather indicate complex stress response networks. This study provides a global overview on the response to different bile acids and only a few selected differentially expressed proteins of this multivariate response could be discussed in more detail at this point. Our comprehensive dataset will be a starting point for future in-depth analyses in which the molecular background and the signal transduction pathways of the bile acid adaptation process of *C. difficile* will be elucidated. This knowledge will be the basis for a development of tailored analogs of bile acids which can efficiently hamper *C. difficile* growth with minimal side effects in patients.

## Author Contributions

SS designed the research. SD, NM, VG, and ST performed the experiments. CW, DZ, and CH were involved in mass spectrometric analyses. NM and RS prepared the samples for electron microscopy and recorded the pictures. DT, NM, and SS analyzed, evaluated and visualized the data. KR and SS wrote the paper.

## Conflict of Interest Statement

The authors declare that the research was conducted in the absence of any commercial or financial relationships that could be construed as a potential conflict of interest.

## Supplementary Material

Supplementary Figures S1, S2, S4 are provided via https://doi.org/10.6084/m9.figshare.7642763. Supplementary Figure S3 is accessed via https://doi.org/10.6084/m9.figshare.7646822.
